# Meta-Analysis Based on Nonconvex Regularization

**DOI:** 10.1038/s41598-020-62473-2

**Published:** 2020-04-01

**Authors:** Hui Zhang, Shou-Jiang Li, Hai Zhang, Zi-Yi Yang, Yan-Qiong Ren, Liang-Yong Xia, Yong Liang

**Affiliations:** 1 Faculty of Information Technology & State Key Laboratory of Quality Research in Chinese Medicines, Macau University of Science and Technology, Taipa, 999078 Macau; 2 School of Mathematics, Northwest University, 710127 Xi’an, China

**Keywords:** Classification and taxonomy, Machine learning

## Abstract

The widespread applications of high-throughput sequencing technology have produced a large number of publicly available gene expression datasets. However, due to the gene expression datasets have the characteristics of small sample size, high dimensionality and high noise, the application of biostatistics and machine learning methods to analyze gene expression data is a challenging task, such as the low reproducibility of important biomarkers in different studies. Meta-analysis is an effective approach to deal with these problems, but the current methods have some limitations. In this paper, we propose the meta-analysis based on three nonconvex regularization methods, which are *L*_1/2_ regularization (meta-Half), Minimax Concave Penalty regularization (meta-MCP) and Smoothly Clipped Absolute Deviation regularization (meta-SCAD). The three nonconvex regularization methods are effective approaches for variable selection developed in recent years. Through the hierarchical decomposition of coefficients, our methods not only maintain the flexibility of variable selection and improve the efficiency of selecting important biomarkers, but also summarize and synthesize scientific evidence from multiple studies to consider the relationship between different datasets. We give the efficient algorithms and the theoretical property for our methods. Furthermore, we apply our methods to the simulation data and three publicly available lung cancer gene expression datasets, and compare the performance with state-of-the-art methods. Our methods have good performance in simulation studies, and the analysis results on the three publicly available lung cancer gene expression datasets are clinically meaningful. Our methods can also be extended to other areas where datasets are heterogeneous.

## Introduction

With the rapid development of biotechnology and its wide applications, many database repositories of high-throughput gene expression data have been created and published. For example, Gene Expression Omnibus (GEO) currently has stored more than 2.76 million samples over 105,000 studies^1^. The gene expression datasets have been widely used in the prediction and diagnosis of diseases, and their application prospects are increasingly promising.

It is desirable to consider variable selection into the analysis of gene expression data due to its small sample size and high dimensionality. Variable selection not only enhances generalization by reducing overfitting, but also enhances interpretability by simplifying the model, i.e., identifying important biomarkers associated with the disease and helping to find the best solution for patients in the treatment process. For a single dataset, there exist many variable selection methods, such as Least Absolute Shrinkage and Selection Operator (LASSO)^2^, *L*_1/2_ regularization^3–5^, Minimax Concave Penalty (MCP)^6^, Smoothly Clipped Absolute Deviation (SCAD)^7–9^, Group LASSO^10^, elastic net^11^, Hard Ridge^12^, SCAD-*L*_2_^13^, Complex Harmonic Regularization (CHR) penalty^14^ and so on. These methods are effective in discovering important biomarkers in a single dataset. However, it is well known that the analysis of gene expression data is still a challenging task due to high noise and low reproducibility of important biomarkers. There are two main reasons for this challenging task. One is that the decisive biomarkers that regulate the phenotypes are usually very sparse compared to the total number of biomarkers in the entire genome, and their effects are usually weak, therefore, the results of individual studies are not remarkable and difficult to reproduce. The other is that the different experimental datasets may come from inconsistent experimental conditions, sample preparation methods, measurement sensitivities or precision, and also from different study groups, biological sample selections. Therefore, the important genes in some studies may be not remarkable in other studies, which we call the data have the heterogeneity. The data heterogeneity reveals the complexity of gene expression data and significantly obstructs gene expression technology in clinical applications.

Since many genomic databases are publicly available, meta-analysis is an effective approach to address the heterogeneity among different datasets and make full use of different datasets. Meta-analysis is a significant technique for clinical diagnosis, which plays an important role in summarizing and synthesizing scientific evidence from multiple studies. Classic meta-analysis methods, which aggregate the summary statistics from individual datasets to obtain total scores and then evaluate them based on statistical significance of all studies, including *p* values^15^, ranks^16,17^, effect sizes^18–21^. Li and Tseng^22^ apply Fisher’s method combining *p* values by summation of log-transformed *p* values, and the method increases the biological interpretation of meta-analysis results. Similar strategies can be applied to combine effect sizes of Random Effects Model (REM) or Fixed Effects Model (FEM) from individual studies. A comprehensive review of these methods is given in the researches^23–25^. These methods perform well in identifying differentially expressed genes, but they ignore the correlations between the covariates (genes). There are some approaches that attempt to model the preprocessed microarray datasets using latent variable-based models^26–30^. In general, latent variables are not observable in the data, but can be inferred from other observed variables. Huo *et al*.^31^ use latent variable to quantify homogeneous and heterogeneous differentially expressed signals across studies to detect genes that are differentially expressed in only a subset of the combined studies. Rashid *et al*.^32^ utilize a penalized Generalized Linear Mixed Model based on latent variable to select gene signatures and address between-study heterogeneity. These methods provide the potential to pool information across genes, making it possible to more clearly infer which genes are differentially expressed. Compared with the previous classical meta-analysis methods, these methods are more complex, which limit their application in practice. Recently, Zhang *et al*.^33^ set different constant terms for multiple studies in the logistic regression model to measure the heterogeneity of the samples. This method assumes that the same variables in multiple studies should make the same contribution to their corresponding responses. In other words, this method conducts variable selection in an ‘all-in-or-all-out’ fashion. In this paper, we consider that some important genes in some studies are likely to be ineffective in other studies, and it is important to allow such flexibility.

Some researchers propose the bi-level selection methods which consider the coefficients of each variable (gene) from all datasets as a group, and simultaneously shrink these groups and the variables within these groups by the penalty function to study the correlation between variables and identify important genes. Existing bi-level selection methods include composite MCP^34^, group Bridge^35^ and group exponential LASSO^36^, meta-SVM^37^ etc. These methods of the aforementioned references generally treat the coefficients of one gene from different datasets as a group, and conduct two levels selection. The first is to determine whether a particular gene is related to the response variable in all datasets, and the second is to determine which dataset contains the identified gene related to the response variable. These methods consider both the heterogeneity and the correlation between the datasets. However, for *M* independent datasets ${\{\left({{\boldsymbol{X}}}_{m},{{\boldsymbol{y}}}_{m}\right)\}}_{m=1}^{M}$, each of which contains *n*_*m*_ samples and *p* variables, these methods consider to solve the problem which has the $\mathop{\sum }\limits_{m=1}^{M}{n}_{m}\times Mp$ dimensional measurement matrix $\widetilde{{\boldsymbol{X}}}=diag({{\boldsymbol{X}}}_{1},{{\boldsymbol{X}}}_{2},\cdots \ ,{{\boldsymbol{X}}}_{M})$, the $\mathop{\sum }\limits_{m=1}^{M}{n}_{m}$ dimensional response $\widetilde{{\boldsymbol{y}}}={\left({{\boldsymbol{y}}}_{1}^{T},{{\boldsymbol{y}}}_{2}^{T},\cdots ,{{\boldsymbol{y}}}_{M}^{T}\right)}^{T}$ and the *Mp* dimensional unknown coefficients ${\boldsymbol{\beta }}={\left({{\boldsymbol{\beta }}}_{1}^{T},{{\boldsymbol{\beta }}}_{2}^{T},\cdots ,{{\boldsymbol{\beta }}}_{M}^{T}\right)}^{T}$, where the superscript *T* represents the transpose of the vector. Since the gene expression data has the characteristics of small sample size and high-dimensional, these methods greatly increase the variable dimension, so it may increase the difficulty of solving the problem.

Zhou and Zhu^38^ propose a new group variable selection method “hierarchical LASSO” that can be used for gene-set selection. The hierarchical LASSO not only removes unimportant groups effectively, but also maintains the flexibility of selecting variables within the group. They also showed that the new method offers the potential for achieving the theoretical “oracle” property. Li *et al*.^39^ propose meta-LASSO for variable selection with high-dimensional meta-analyzed data. The meta-LASSO not only improves the ability to identify important genes with the strength of multiple datasets, but also maintains the flexibility of selection between datasets to consider the data heterogeneity.

For many practical applications, LASSO often cannot find the most sparse solutions (this is extremely important for model selection), and it is inefficient when the errors in data have heavy tail distribution^2^. Zhao and Yu^40^ give the Strong Irrepresentable Condition for the model selection consistency of LASSO, and show that to induce sparsity, LASSO shrinks the estimates for the nonzero coefficients too heavily. When Strong Irrepresentable Condition fails, the irrelevant covariates are correlated with the relevant covariates enough to be picked up by LASSO to compensate the over-shrinkage of the nonzero parameters. Therefore, to get universal consistency, some nonconvex regularization methods have been proposed in recent years, such as *L*_1/2_ penalty, Minimax Concave Penalty (MCP) and Smoothly Clipped Absolute Deviation (SCAD) penalty etc. These methods achieve both selection consistency and nearly unbiasedness, which make them widely applied in signal/image processing, statistics and machine learning, such as biological feature selection^14,41–44^, compressed sensing and low rank matrix completion^8,45–48^, sparse signals separation and image inpainting^49,50^, and dictionary learning^51^ etc.

In this paper, we propose the meta-analysis based on three nonconvex regularization methods (*L*_1/2_ regularization, MCP regularization and SCAD regularization), dubbed as meta-Half, meta-MCP and meta-SCAD respectively. Our methods combine the advantages of meta-analysis and the nonconvex regularization methods. We propose the efficient algorithms which apply the nonconvex iterative thresholding algorithms based on approximate message passing (Half-AMP, MCP-AMP and SCAD-AMP)^52,53^ to solve our models. Furthermore, we apply our methods to the simulation data and three publicly available lung cancer gene expression datasets, and compare the performance of our methods with other four state-of-the-art methods, which are meta-LASSO, composite MCP, group Bridge and group exponential LASSO. The experiments results show that our methods have favorable performance.

## Methodology

In this section, we study the meta-analysis based on the three nonconvex regularization methods (*L*_1/2_ regularization, MCP regularization and SCAD regularization).

Consider *M* independent datasets $D={\{\left({{\boldsymbol{X}}}_{m},{{\boldsymbol{y}}}_{m}\right)\}}_{m=1}^{M}$, each of which contains *n*_*m*_ samples. Denote ${{\boldsymbol{X}}}_{m}={({{\boldsymbol{x}}}_{m1},{{\boldsymbol{x}}}_{m2},\cdots ,{{\boldsymbol{x}}}_{m,{n}_{m}})}^{T}$ and ${{\boldsymbol{y}}}_{m}={({y}_{m1},{y}_{m2},\cdots ,{y}_{m,{n}_{m}})}^{T}$, where the superscript *T* represents the transpose of the vector, ${{\boldsymbol{x}}}_{mi}={({x}_{mi,1},{x}_{mi,2},\cdots ,{x}_{mi,p})}^{T}$ (*i* = 1, 2, ⋯, *n*_*m*_) is *i*th sample in the *m*th dataset which contains *p* variables (genes), and *y*_*mi*_ is the response variable, in this paper, we consider the response variable is a binary phenotype (for example, if the *i*th sample of the *m*th dataset is a disease patient, *y*_*mi*_ is 1, and 0 otherwise). The *p* genes are assumed common in all datasets. We assume the conditional probability that *y*_*mi*_ takes value 1 given the gene expression vector ***x***_*mi*_ follows the logistic regression model

1 $${\rm{\log }}\,\frac{Pr\left({y}_{mi}=1| {{\boldsymbol{x}}}_{mi}\right)}{Pr\left({y}_{mi}=0| {{\boldsymbol{x}}}_{mi}\right)}={\beta }_{m0}+{{\boldsymbol{x}}}_{mi}^{T}{{\boldsymbol{\beta }}}_{m},i=1,2,\cdots \ ,{n}_{m},\,m=1,2,\cdots \ ,M,$$

where *β*_*m*0_ is an intercept and ${{\boldsymbol{\beta }}}_{m}={\left({\beta }_{m1},\cdots ,{\beta }_{mp}\right)}^{T}$ is the unknown coefficients for the *m*th data. Due to heterogeneity between datasets, we allow *β*_*m*0_ and *β*_*m*_ in (1) to vary with *m*. We hope to find the true nonzero components of *β*_*m*_ for each dataset.

Compared with the variable selection of single dataset model, the variable selection of the *M* datasets models are distinguishing and peculiar. On the one hand, each variable has *M* coefficients, which belong to the same explanatory variable. Therefore, there is some correlation or similarity, which makes it impossible to make coefficient estimation and variable selection separately, otherwise this correlation will be ignored. On the other hand, the significance of variables is not identical, so we cannot simply synthesize estimation. The penalization methods with meta-analysis make full use of this particularity to study data differences. These methods conduct variable selection by maximizing,

2 $$\mathop{\sum }\limits_{m=1}^{M}{\ell }_{m}({\beta }_{m0},{{\boldsymbol{\beta }}}_{m})-P({\boldsymbol{\beta }};\lambda ),$$

where *ℓ*_*m*_(*β*_*m*0_, **β**_*m*_) is the log-likelihood for the *m*th dataset and has the following form

$${\ell }_{m}({\beta }_{m0},{{\boldsymbol{\beta }}}_{m})=\mathop{\sum }\limits_{i=1}^{{n}_{m}}[{y}_{mi}({\beta }_{m0}+{{\boldsymbol{x}}}_{mi}^{T}{{\boldsymbol{\beta }}}_{m})-\log \{1+\exp ({\beta }_{m0}+{{\boldsymbol{x}}}_{mi}^{T}{{\boldsymbol{\beta }}}_{m})\}],$$

*P* is a penalty function and *λ* is the regularization parameter that controls the complexity of the machine.

In this paper, we focus on the three nonconvex regularization methods (*L*_1/2_ regularization, MCP regularization and SCAD regularization), and through the hierarchical decomposition of coefficients that maintain the flexibility of variable selection as well incorporate the relationship between different datasets. We consider the following hierarchical reparameterization:

3 $${\beta }_{mj}={h}_{j}{\xi }_{mj},m=1,2,\cdots \ ,M;j=1,2,\cdots \ ,p.$$

The parameter *h*_*j*_ is the effect of the *j*th gene, and the different *m* for *ξ*_*mj*_ reflects the different effects of the *j*th gene among *M* datasets. If *h*_*j*_ = 0, then ${{\boldsymbol{\beta }}}_{j}={\left({\beta }_{1j},{\beta }_{2j},\cdots ,{\beta }_{Mj}\right)}^{T}={\bf{0}}$, this indicates that the *j*th gene is not significant in all *M* datasets. If *h*_*j*_ ≠ 0, then whether the *β*_*mj*_ is equal to 0 depends on whether *ξ*_*mj*_ is equal to 0. Since the *M* datasets may have heterogeneity (the *M* datasets may come from inconsistent experimental conditions, sample preparation methods, measurement sensitivities or precision, and also from different study groups, biological sample selections.), then one gene is important in some datasets may be not remarkable in other datasets. Through *ξ*_*mj*_ contral *β*_*mj*_ to keep the selection flexibility among *M* datasets. If the *M* datasets have no heterogeneity, then *h*_*j*_ = *β*_*mj*_ for *m* = 1, ⋯, *M* defined in (2) and *ξ*_*mj*_ = 1 for all *j* and *m*. With reparameterization (3), we propose a meta-analysis method based on nonconvex regularization. Our method selects important genes by solving

4 $$\mathop{\max }\limits_{{{\boldsymbol{\beta }}}_{0},{\boldsymbol{h}},{\boldsymbol{\xi }}}\mathop{\sum }\limits_{m=1}^{M}{\ell }_{m}({\beta }_{m0},{\boldsymbol{h}},{{\boldsymbol{\xi }}}_{m})-\mathop{\sum }\limits_{j=1}^{p}P({h}_{j};{\lambda }_{h})-\mathop{\sum }\limits_{j=1}^{p}\mathop{\sum }\limits_{m=1}^{M}P({\xi }_{mj};{\lambda }_{\xi }),$$

where *ℓ*_*m*_(*β*_*m*0_, ***h***, **ξ**_*m*_) is the likelihood function and has the following form

5 $${\ell }_{m}({\beta }_{m0},{\boldsymbol{h}},{{\boldsymbol{\xi }}}_{m})=\mathop{\sum }\limits_{i=1}^{{n}_{m}}[{y}_{mi}({\beta }_{m0}+{{\boldsymbol{x}}}_{mi}^{T}({\boldsymbol{h}}\cdot {{\boldsymbol{\xi }}}_{m}))-\log \{1+\exp ({\beta }_{m0}+{{\boldsymbol{x}}}_{mi}^{T}({\boldsymbol{h}}\cdot {{\boldsymbol{\xi }}}_{m}))\}],$$

${\boldsymbol{h}}={\left({h}_{1},{h}_{2},\cdots ,{h}_{p}\right)}^{T}$, ${{\boldsymbol{\xi }}}_{m}={({\xi }_{m1},{\xi }_{m1},\cdots ,{\xi }_{mp})}^{T}$, ${{\boldsymbol{\beta }}}_{0}={({\beta }_{10},{\beta }_{20},\cdots ,{\beta }_{M0})}^{T}$, ${\boldsymbol{\xi }}={\left({{\boldsymbol{\xi }}}_{1}^{T},{{\boldsymbol{\xi }}}_{2}^{T},\cdots ,{{\boldsymbol{\xi }}}_{M}^{T}\right)}^{T}$, and ***h*** ⋅ **ξ**_*m*_ means the element-wise product. *P*( ⋅ ) is a nonconvex penalty function. In this paper, considering the three nonconvex penalty function, the *L*_1/2_ penalty, the MCP penalty and the SCAD penalty. The *L*_1/2_ penalty function is ${P}_{{L}_{1/2}}(x;\lambda )=\lambda \parallel x{\parallel }_{1/2}^{1/2}=\mathop{\sum }\limits_{i=1}^{p}| {x}_{i}{| }^{1/2}$. MCP penalty function has the following form

$${P}_{MCP}(x;\lambda )=\lambda {\int }_{0}^{x}{\left(1-\frac{s}{\gamma \lambda }\right)}_{+}ds,$$

where ${\left(1-\frac{s}{\gamma \lambda }\right)}_{+}=\max \left\{1-\frac{s}{\gamma \lambda },0\right\}$. The SCAD penalty function has the following form

$${P}_{SCAD}(x;\lambda )=\lambda | x| {I}_{\{0\le | x|  < \lambda \}}+\left(\frac{(a-1){\lambda }^{2}}{2}+{\lambda }^{2}\right){I}_{\{| x| \ge a\lambda \}}+\left(\frac{a\lambda (| x| -\lambda )-\left(| x{| }^{2}-{\lambda }^{2}\right)/2}{a-1}+{\lambda }^{2}\right){I}_{\{\lambda \le | x|  < a\lambda \}},$$

we call these three nonconvex penalties for the methods (4) as “meta-Half”, “meta-MCP” and “meta-SCAD”, respectively.

## Algorithm

In this section, we give the efficient algorithms (Algorithm 1) to solve our models. Note that we can assume that the mean of the predictor variable is zero (through the location transformation). (4) can be decomposed into two nonconvex problems, each of which views ***h*** or **ξ** as fixed. We propose to iteratively solve **β**_0_, ***h***, and **ξ** in (4). First, we fix **β**_0_ and **ξ** in (4) to maximize ***h***. We next fix **β**_0_ and ***h*** to maximize **ξ**. Finally, we maximize over **β**_0_ by fixing ***h*** and **ξ**. Iterate these steps until the algorithm converges. Since at each step, the value of the objective function (4) decreases, the solution is guaranteed to converge. Specifically, the algorithm is described as follows

**Algorithm 1:**
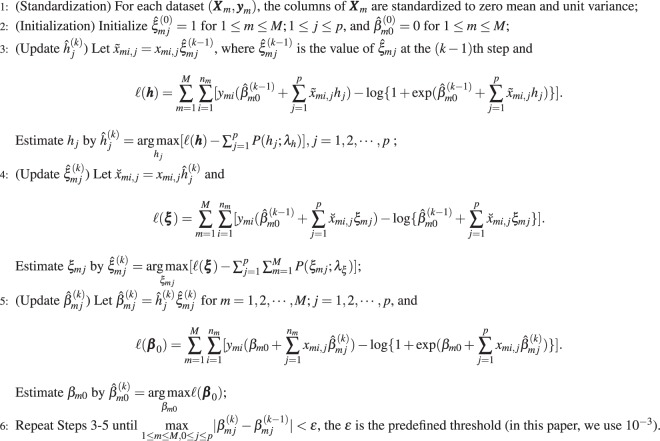
The iterative optimization algorithm for solving our meta-analysis based on nonconvex regularization models.

Step 3 and step 4 are general nonconvex regularization problem. We^52,53^ propose the nonconvex iterative thresholding algorithms based on approximate message passing (Half-AMP, MCP-AMP and SCAD-AMP) to solve the nonconvex regularization problem, and verified the effectiveness of the algorithms through theoretical analysis and experiment. In this paper, for the two problems in step 3 and step 4, we apply the Half-AMP algorithm, the MCP-AMP algorithm and the SCAD-AMP algorithm to solve the meta-Half, meta-MCP and meta-SCAD respectively.

In order to solve the above two problems in step 3 and step 4, we first consider the solution of the traditional logistic regression model. Here we omit the intercept term (in fact, just rewrite the input variable as ${\widetilde{{\boldsymbol{x}}}}_{i}={\left(1,{{\boldsymbol{x}}}_{i}^{T}\right)}^{T}$), the logistic regression can be expressed as the following optimization problem

6 $$\widehat{{\boldsymbol{\beta }}}=\mathop{\arg \min }\limits_{{\boldsymbol{\beta }}\in {{\mathbb{R}}}^{p+1}}\ell ({\boldsymbol{\beta }})=\mathop{\arg \min }\limits_{{\boldsymbol{\beta }}\in {{\mathbb{R}}}^{p+1}}\left\{-\mathop{\sum }\limits_{i=1}^{N}\left[{y}_{i}\left({{\boldsymbol{x}}}_{i}^{{\rm{T}}}{\boldsymbol{\beta }}\right)-{\rm{ln}}\,\left(1+\exp \left({{\boldsymbol{x}}}_{i}^{{\rm{T}}}{\boldsymbol{\beta }}\right)\right)\right]\right\}.$$

Differentiating *ℓ*(**β**) with respect to **β**, we can get

7 $$\frac{\partial \ell ({\boldsymbol{\beta }})}{\partial {\boldsymbol{\beta }}}=-\mathop{\sum }\limits_{i=1}^{N}{{\boldsymbol{x}}}_{i}\left({y}_{i}-\mu \left({{\boldsymbol{x}}}_{i};{\boldsymbol{\beta }}\right)\right),$$

where $\mu \left({{\boldsymbol{x}}}_{i};{\boldsymbol{\beta }}\right)=\frac{\exp \left({{\boldsymbol{x}}}_{i}^{{\rm{T}}}{\boldsymbol{\beta }}\right)}{1+\exp \left({{\boldsymbol{x}}}_{i}^{{\rm{T}}}{\boldsymbol{\beta }}\right)}.$ To find the optimal solution $\widehat{{\boldsymbol{\beta }}}$ of equation (6), we let $\frac{\partial \ell ({\boldsymbol{\beta }})}{\partial {\boldsymbol{\beta }}}=0$, and use the Newton-Raphson iteration algorithm which requires computing the second derivative, the Hessian matrix has the following form

8 $$\frac{{\partial }^{2}\ell ({\boldsymbol{\beta }})}{\partial {\boldsymbol{\beta }}\partial {{\boldsymbol{\beta }}}^{{\rm{T}}}}=\mathop{\sum }\limits_{i=1}^{N}{{\boldsymbol{x}}}_{i}{{\boldsymbol{x}}}_{i}^{{\rm{T}}}\mu \left({{\boldsymbol{x}}}_{i};{\boldsymbol{\beta }}\right)\left(1-\mu \left({{\boldsymbol{x}}}_{i};{\boldsymbol{\beta }}\right)\right).$$

Hence, given the current estimated value **β**^old^ of **β**, the new estimated value **β**^new^ is updated as following

9 $${{\boldsymbol{\beta }}}^{{\rm{new}}}={{\boldsymbol{\beta }}}^{{\rm{old}}}-{\left(\frac{{\partial }^{2}\ell ({\boldsymbol{\beta }})}{\partial {\boldsymbol{\beta }}\partial {{\boldsymbol{\beta }}}^{{\rm{T}}}}\right)}^{-1}\frac{\partial \ell ({\boldsymbol{\beta }})}{\partial {\boldsymbol{\beta }}},$$

where the value of the derivative (and second derivative) is calculated at the point **β**^old^. The equation (9) can be expressed by matrix form. Let ***X*** be a *N* × *P* matrix, where the *i*-th row is ***x***_*i*_; *W* is a diagonal matrix, and the elements on the diagonal

10 $${w}_{i}=\mu \left({{\boldsymbol{x}}}_{i};{\boldsymbol{\beta }}\right)\left(1-\mu \left({{\boldsymbol{x}}}_{i};{\boldsymbol{\beta }}\right)\right).$$

Let ${\boldsymbol{y}}={\left({y}_{1},{y}_{2},\cdots ,{y}_{N}\right)}^{{\rm{T}}}$, ${\boldsymbol{\mu }}={\left(\mu \left({{\boldsymbol{x}}}_{1};{\boldsymbol{\beta }}\right),\mu \left({{\boldsymbol{x}}}_{2};{\boldsymbol{\beta }}\right),\cdots ,\mu \left({{\boldsymbol{x}}}_{N};{\boldsymbol{\beta }}\right)\right)}^{{\rm{T}}},$ then the formulas (7) and (8) can be expressed as

11 $$\frac{\partial \ell ({\boldsymbol{\beta }})}{\partial {\boldsymbol{\beta }}}=-{{\boldsymbol{X}}}^{{\rm{T}}}({\boldsymbol{y}}-{\boldsymbol{\mu }}),\quad \frac{{\partial }^{2}\ell ({\boldsymbol{\beta }})}{\partial {\boldsymbol{\beta }}\partial {{\boldsymbol{\beta }}}^{{\rm{T}}}}={{\boldsymbol{X}}}^{{\rm{T}}}{\boldsymbol{WX}}.$$

Therefore, Newton-Raphson iteration (9) can be expressed as

12 $$\begin{array}{lll}{{\boldsymbol{\beta }}}^{{\rm{new}}} & = & {{\boldsymbol{\beta }}}^{{\rm{old}}}+{({{\boldsymbol{X}}}^{{\rm{T}}}{\boldsymbol{WX}})}^{-1}{{\boldsymbol{X}}}^{{\rm{T}}}({\boldsymbol{y}}-{\boldsymbol{\mu }})\\  & = & {({{\boldsymbol{X}}}^{{\rm{T}}}{\boldsymbol{WX}})}^{-1}{{\boldsymbol{X}}}^{{\rm{T}}}{\boldsymbol{W}}\left({\boldsymbol{X}}{{\boldsymbol{\beta }}}^{{\rm{old}}}+{{\boldsymbol{W}}}^{-1}({\boldsymbol{y}}-{\boldsymbol{\mu }})\right)\\  & = & {({{\boldsymbol{X}}}^{{\rm{T}}}{\boldsymbol{WX}})}^{-1}{{\boldsymbol{X}}}^{{\rm{T}}}{\boldsymbol{Wz}},\end{array}$$

where

13 $${\boldsymbol{z}}={\boldsymbol{X}}{{\boldsymbol{\beta }}}^{{\rm{old}}}+{{\boldsymbol{W}}}^{-1}({\boldsymbol{y}}-{\boldsymbol{\mu }}).$$

It can be seen that each Newton-Raphson iteration actually solves the weighted least squares problem as follows

14 $$\widehat{{\boldsymbol{\beta }}}=\mathop{\arg \min }\limits_{{\boldsymbol{\beta }}}{({\boldsymbol{z}}-{\boldsymbol{X}}{\boldsymbol{\beta }})}^{{\rm{T}}}{\boldsymbol{W}}({\boldsymbol{z}}-{\boldsymbol{X}}{\boldsymbol{\beta }}).$$

Based on the solution process of the traditional logistic regression model, a similar iterative algorithm can be used to solve the logistic regression with nonconvex penalties problem, and only a slight deformation of the formula (14) is needed to obtain the iterative algorithm.

15 $$\widehat{{\boldsymbol{\beta }}}=\mathop{\arg \min }\limits_{{\boldsymbol{\beta }}}{({\boldsymbol{z}}-{\boldsymbol{X}}{\boldsymbol{\beta }})}^{{\rm{T}}}{\boldsymbol{W}}({\boldsymbol{z}}-{\boldsymbol{X}}{\boldsymbol{\beta }})+P({\boldsymbol{\beta }};\lambda ),$$

where *P*( ⋅ ; ⋅ ) is the nonconvex penalty function. It is easy to see that the minimization problem (15) is equivalent to the maximization problem (2). The minimization problem (15) can be solved by the nonconvex iterative thresholding algorithms based on approximate message passing^52,53^ (which are based on linear regression ***y*** = ***X*β**, ***y*** ∈ *R*^*N*^, ***X*** ∈ *R*^*N*×*p*^). The algorithms are according to the following iteration:

16 $${{\boldsymbol{\beta }}}^{(k+1)}=\eta \left({{\boldsymbol{\beta }}}^{(k)}+{{\boldsymbol{X}}}^{T}{{\boldsymbol{r}}}^{(k)}\right),$$

17 $${{\boldsymbol{r}}}^{(k+1)}={\boldsymbol{y}}-{\boldsymbol{X}}{{\boldsymbol{\beta }}}^{(k)}+\frac{1}{\delta }{{\boldsymbol{r}}}^{(k)}\langle \eta {\prime} ({{\boldsymbol{\beta }}}^{(k-1)}+{{\boldsymbol{X}}}^{T}{{\boldsymbol{r}}}^{(k-1)})\rangle ,$$

where $\delta =\frac{p}{N}$ represents a measure of indeterminacy of the measurement system, in this paper, considering the case *δ* is fixed for *N* → *∞*. For a vector *u* = (*u*_1_, *u*_2_, . . . , *u*_*N*_), $\langle u\rangle ={\sum }_{i=1}^{N}{u}_{i}$/*N*, $\eta {\prime} (x)=\frac{\partial }{\partial x}\eta (x)$. *η* is the thresholding function, in this paper, *η* represents the Half thresholding function, the MCP thresholding function and the SCAD thresholding function, respectively. The Half thresholding function is

18 $$\eta (u;\lambda )=\{\begin{array}{cc}g(u;\lambda ), & |u| > \lambda ,\\ 0, & |u|\le \lambda ,\end{array}$$

where

$$\left.g(u;\lambda )=\frac{2}{3}u\left(1+\cos \left(\frac{2\pi }{3}-\frac{2}{3}h(u;\lambda )\right)\right),h(u;\lambda )=\arccos \left(\frac{\sqrt{2}}{2}{\left(\frac{\lambda }{| u| }\right)}^{\frac{2}{3}}\right)\right).$$

The MCP thresholding function is

$${\eta }_{MCP}(u;\lambda )=\left\{\begin{array}{ll}u, & | u|  > \gamma \lambda ,\\ \frac{{\eta }^{S}(u;\lambda )}{1-\frac{1}{\gamma }}, & | u| \le \gamma \lambda ,\end{array}\right.$$

where

$${\eta }^{S}(u;\lambda )=\left\{\begin{array}{ll}u-sign(u)\lambda , & | u|  > \lambda ,\\ 0, & | u| \le \lambda ,\end{array}\right.$$

where *sign*(*u*) is sign function,

$$sign(u)=\left\{\begin{array}{ll}1, & u > 0,\\ -1, & u < 0,\\ 0, & u=0.\end{array}\right.$$

The SCAD thresholding function is

$${\eta }_{SCAD}(u;\lambda )=\left\{\begin{array}{ll}u, & | u|  > a\lambda ,\\ \frac{(a-1)u-sign(u)a\lambda }{a-2}, & 2\lambda  < | u| \le a\lambda ,\\ u-sign(u)\lambda , & \lambda  < | u| \le 2\lambda ,\\ 0, & otherwise.\end{array}\right.$$

## Theoretical Properties

In this section, we study the theoretical properties of the meta-Half method. The meta-Half has the following uniform form

19 $$\mathop{\max }\limits_{{{\boldsymbol{\beta }}}_{0},{\boldsymbol{h}},{\boldsymbol{\xi }}}\mathop{\sum }\limits_{m=1}^{M}{\ell }_{m}({\beta }_{m0},{\boldsymbol{h}},{{\boldsymbol{\xi }}}_{m})-{\lambda }_{h}\mathop{\sum }\limits_{j=1}^{p}| {h}_{j}{| }^{\frac{1}{2}}-{\lambda }_{\xi }\mathop{\sum }\limits_{j=1}^{p}\mathop{\sum }\limits_{m=1}^{M}| {\xi }_{mj}{| }^{\frac{1}{2}},$$

there are two tuning parameters *λ*_*h*_ and *λ*_*ξ*_ in (19), we first show that the two tuning parameters can be simplified into one. Specifically, let *λ* = *λ*_*h*_*λ*_*ξ*_, we can show that (19) is equivalent to

20 $$\mathop{\max }\limits_{{{\boldsymbol{\beta }}}_{0},{\boldsymbol{h}},{\boldsymbol{\xi }}}\mathop{\sum }\limits_{m=1}^{M}{\ell }_{m}({\beta }_{m0},{\boldsymbol{h}},{{\boldsymbol{\xi }}}_{m})-\mathop{\sum }\limits_{j=1}^{p}| {h}_{j}{| }^{\frac{1}{2}}-\lambda \mathop{\sum }\limits_{j=1}^{p}\mathop{\sum }\limits_{m=1}^{M}| {\xi }_{mj}{| }^{\frac{1}{2}}.$$

### Lemma 1.

*If* $\left({\widetilde{{\boldsymbol{\beta }}}}_{0},\widetilde{{\boldsymbol{h}}},\widetilde{{\boldsymbol{\xi }}}\right)$*is a local maximizer of* (19). *Then there exists a local maximizer* $({\hat{{\boldsymbol{\beta }}}}_{0},\hat{{\boldsymbol{h}}},\hat{{\boldsymbol{\xi }}})$ *of* (20) *such that* ${\widetilde{h}}_{j}{\widetilde{\xi }}_{mj}={ {\hat{h}} }_{j}{\widehat{\xi }}_{mj}$ *and* ${\widetilde{{\boldsymbol{\beta }}}}_{0}={\widehat{{\boldsymbol{\beta }}}}_{0}$. *Vice versa*.

The proof is in the Supplementary. This lemma indicates that although (19) and (20) may provide different *h*_*j*_ and *ξ*_*mj*_, the final fitted models from them are the same. Therefore, we only need to tune one parameter *λ* = *λ*_*h*_*λ*_*ξ*_ other than tune *λ*_*h*_ and *λ*_*ξ*_ separately in practice.

We then show that (20) can also be written in an equivalent form using the original regression coefficients *β*_*mj*_.

### Lemma 2.

*Suppose* $\left(\widehat{{\boldsymbol{h}}},\widehat{{\boldsymbol{\xi }}}\right)$ *is a local maximizer of* (20), *for* *j* = 1, 2, ⋯, *p*, *let* ${\widehat{\beta }}_{mj}={ {\hat{h}} }_{j}{\widehat{\xi }}_{mj}$, ${\widehat{{\boldsymbol{\beta }}}}_{j}={({\widehat{\beta }}_{1j},{\widehat{\beta }}_{2j},\cdots ,{\widehat{\beta }}_{Mj})}^{T}$ *and* ${\widehat{{\boldsymbol{\xi }}}}_{j}={({\widehat{\xi }}_{1j},{\widehat{\xi }}_{2j},\cdots ,{\widehat{\xi }}_{Mj})}^{T}$,

(*a*) *If* $${ {\hat{h}} }_{j}=0$$, *then* $${\widehat{{\boldsymbol{\beta }}}}_{j}={\bf{0}}$$;

(*b*) *If* ${ {\hat{h}} }_{j}\ne 0$, *then* ${\widehat{{\boldsymbol{\beta }}}}_{j}\ne {\bf{0}}$ *and* ${ {\hat{h}} }_{j}=\lambda \parallel {\widehat{{\boldsymbol{\beta }}}}_{j}{\parallel }_{1/2}^{1/2}$, ${\widehat{{\boldsymbol{\xi }}}}_{j}=\frac{{\widehat{{\boldsymbol{\beta }}}}_{j}}{\lambda \parallel {\widehat{{\boldsymbol{\beta }}}}_{j}{\parallel }_{1/2}^{1/2}}$.

The proof is in the Supplementary.

### Theorem 1.

*If* $({\hat{{\boldsymbol{\beta }}}}_{0},\hat{{\boldsymbol{h}}},\hat{{\boldsymbol{\xi }}})$ *is a local maximizer of* (20), *then* $\widehat{{\boldsymbol{\beta }}}$ *with* ${\widehat{\beta }}_{mj}={ {\hat{h}} }_{j}{\widehat{\xi }}_{mj}$, *is a local maximizer of*

21 $${\max }_{{\boldsymbol{\beta }}}\mathop{\sum }\limits_{m=1}^{M}{\ell }_{m}({{\boldsymbol{\beta }}}_{m})-\lambda \mathop{\sum }\limits_{j=1}^{p}\mathop{\sum }\limits_{m=1}^{M}| {\beta }_{mj}{| }^{\frac{1}{2}},$$

*where* ${\boldsymbol{\beta }}={({\beta }_{10},{\beta }_{11},\cdots ,{\beta }_{Mp})}^{T}$. *On the other hand, if* $\widehat{{\boldsymbol{\beta }}}$ *is a solution of* (21), *then* $({\hat{{\boldsymbol{\beta }}}}_{0},\hat{{\boldsymbol{h}}},\hat{{\boldsymbol{\xi }}})$ *is a solution of* (20), *where* ${\widehat{{\boldsymbol{\beta }}}}_{0}={\left({\widehat{\beta }}_{10},{\widehat{\beta }}_{20},\cdots ,{\widehat{\beta }}_{M0}\right)}^{T}$, $\parallel {\widehat{{\boldsymbol{\beta }}}}_{j}{\parallel }_{1/2}^{1/2}=\mathop{\sum }\limits_{m=1}^{M}| {\widehat{\beta }}_{mj}{| }^{\frac{1}{2}}$,

$$(\widehat{{\boldsymbol{h}}},\widehat{{\boldsymbol{\xi }}})=\left\{\begin{array}{ll}{ {\hat{h}} }_{j}=0,{\widehat{{\boldsymbol{\xi }}}}_{j}={\bf{0}}, & if\,{\widehat{{\boldsymbol{\beta }}}}_{j}={\bf{0}},\\ { {\hat{h}} }_{j}=\lambda \parallel {\widehat{{\boldsymbol{\beta }}}}_{j}{\parallel }_{1/2}^{1/2},{\widehat{{\boldsymbol{\xi }}}}_{j}=\frac{{\widehat{{\boldsymbol{\beta }}}}_{j}}{\lambda \parallel {\widehat{{\boldsymbol{\beta }}}}_{j}{\parallel }_{1/2}^{1/2}}, & if\,{\widehat{{\boldsymbol{\beta }}}}_{j}\ne {\bf{0}},\end{array}\right.$$

*where* $$\widehat{{\boldsymbol{h}}}=({ {\hat{h}} }_{1},{ {\hat{h}} }_{2},\cdots \ ,{ {\hat{h}} }_{p})$$, $$\widehat{{\boldsymbol{\xi }}}={({\widehat{\xi }}_{11},{\widehat{\xi }}_{12},\cdots ,{\widehat{\xi }}_{Mp})}^{T}$$, $${\widehat{{\boldsymbol{\beta }}}}_{j}={({\widehat{\beta }}_{1j},{\widehat{\beta }}_{2j},\cdots ,{\widehat{\beta }}_{Mj})}^{T}$$ *and* $${\widehat{{\boldsymbol{\xi }}}}_{j}={({\widehat{\xi }}_{1j},{\widehat{\xi }}_{2j},\cdots ,{\widehat{\xi }}_{Mj})}^{T}$$.

The proof is in the Supplementary. If we regard one gene’s effects among all datasets as a “group”, then (21) imposes an *L*_1_ penalty on each group and a square root penalty on individual elements within a group. The following theorem shows the theoretical properties of the meta-Half.

### Theorem 2.

*The meta-Half method possesses sparsity, unbiasedness and oracle properties*.

The proof is in the Supplementary.

## Experiments

In this section, we analyze the performance of our methods (meta-Half, meta-MCP and meta-SCAD) by simulation and real-data analysis. We compare these three methods with other four methods, which are meta-LASSO, composite MCP, group Bridge and group exponential LASSO. The codes of our methods are available at GitHub (https://github.com/zhhui019/meta-nonconvex). The meta-LASSO is implemented by Li *et al*.^39^. The composite MCP, the group Bridge and the group exponential LASSO are implemented by Patrick Breheny and Yaohui Zeng’s R package “grpreg”.

### Simulations

Simulation studies are performed to compare the performance of the proposed meta-Half, meta-MCP and meta SCAD with the meta-LASSO, composite MCP, group Bridge and group exponential LASSO.

#### Generate simulated data

In this simulation, we use the normal distribution to generate the gene expression ***x***_*mi*_ (*m* = 1, 2, ⋯, *M*; *i* = 1, 2, ⋯, *n*_*m*_) with *M* = 10 datasets, each dataset contains *n*_*m*_ = 50 samples, and each sample contains *p* = 1, 000 genes. The response *y*_*mi*_ is generated from a logistic model

$$Pr\left({y}_{mi}=1| {{\boldsymbol{x}}}_{mi}\right)=\frac{\exp \left({{\boldsymbol{x}}}_{mi}^{T}{{\boldsymbol{\beta }}}_{m}^{\ast }\right)}{[1+\exp ({{\boldsymbol{x}}}_{mi}^{T}{{\boldsymbol{\beta }}}_{m}^{\ast })]},$$

where ${{\boldsymbol{\beta }}}_{m}^{\ast }=({\beta }_{m1}^{\ast },{\beta }_{m2}^{\ast },\cdots \ ,{\beta }_{mp}^{\ast })$ and we suppose that the intercept term ${\beta }_{m0}^{\ast }=0.$ We let ${\beta }_{mj}^{\ast }={\alpha }_{mj}{\theta }_{mj}$ simulate possible data heterogeneity, for *m* = 1, 2 ⋯, *M*; *j* = 1, 2 ⋯, 10, *α*_*mj*_ are generated from *N*(3, 0. 5^2^) and *θ*_*mj*_ are generated from Bernoulli(*π*_0_), for *m* = 1, 2 ⋯, *M*; *j* = 11, 12 ⋯, 1000, let ${\beta }_{mj}^{\ast }=0$. This means that the first 10 genes of each dataset are important to the response with probability *π*_0_. The value *α*_*mj*_ demonstrates whether the *j*th gene is important in the *m*th dataset, and the value *θ*_*mj*_ demonstrates different levels of heterogeneity among different datasets, in this simulation, considering *π*_0_ = 0.9, 0.5, 0.2 to represent the low, medium and high heterogeneity. We run 30 replicates and report the average measurement.

For the all methods, the tuning parameters are selected by minimizing the BIC:

22 $$BIC(\lambda )=\mathop{\sum }\limits_{m=1}^{M}\left\{-2{\ell }_{m}({\widehat{{\boldsymbol{\beta }}}}_{m,\lambda })+{S}_{m}{\rm{\log }}\,({n}_{m})\right\},$$

where ${\widehat{{\boldsymbol{\beta }}}}_{m,\lambda }$ is the estimated coefficients in the *m*th dataset, *λ* is the tuning parameter, *S*_*m*_ is the number of non-zero elements of ${\widehat{{\boldsymbol{\beta }}}}_{m,\lambda }$, ${\ell }_{m}({\widehat{{\boldsymbol{\beta }}}}_{m,\lambda })$ is the log-likelihood for the *m*th dataset and has the form (5).

#### Analysis of simulation

The variable selection performance of the seven methods is evaluated using the selection sensitivity, specificity and accuracy of coefficient **β**. The sensitivity is the proportion of non-zero ${\beta }_{mj}^{\ast }$’s that are correctly estimated as non-zero, the specificity is the proportion of zero ${\beta }_{mj}^{\ast }$’s that are correctly estimated as zero and the accuracy is the proportion of ${\beta }_{mj}^{\ast }$’s that are correctly estimated.

**Figure 1:**
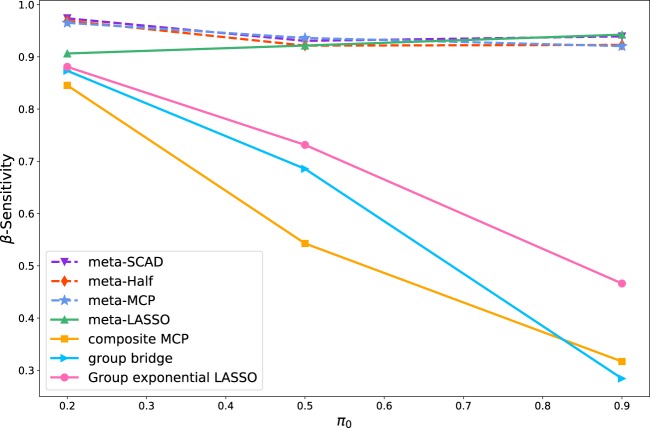
The sensitivity trend of coefficient **β** for all seven methods with the varying levels of heterogeneity.

The simulation results are summarized in Table 1 (The variable selection performance of the seven methods are evaluated using the selection sensitivity, specificity and accuracy of coefficient **β**). Table 1 shows that the specificity and accuracy of the coefficients **β** of all seven methods are similar. The sensitivity trend of coefficient **β** for all seven methods with the varying levels of heterogeneity is shown in Fig. 1. Figure 1 shows that the sensitivity (the proportion of non-zero ${\beta }_{mj}^{\ast }$’s that are correctly estimated as non-zero) of the composite MCP, the group Bridge and the group exponential LASSO dramatically decreases as *π*_0_ increases, while the sensitivity of meta-Half, meta-MCP, meta-SCAD and meta-LASSO remains above 0.9 for *π*_0_ = 0.2, 0.5, 0.9. When *π* = 0.2, the sensitivity of meta-Half, meta-MCP and meta-SCAD are 0.9693, 0.9651 and 0.9738, respectively, which are significantly higher than other methods. This result shows that our proposed meta-Half, meta-MCP and meta-SCAD have the superior performance when data heterogeneity is strong (*π*_0_ is small). With the weakening of data heterogeneity(*π* = 0.5, 0.9), the performance of the four meta methods (meta-Half, meta-MCP, meta-SCAD and meta-LASSO) tends to be comparable. The specificity and accuracy of the coefficients for all seven methods are similar.

**Table 1:** The sensitivity, specificity and accuracy of coefficient **β** of the seven methods: presented values are the mean (standard error).

		***π*** = 0.2	***π*** = 0.5	***π*** = 0.9
meta-Half	Sensitivity	0.9693 (1.70*E* − 03)	0.9215 (4.97*E* − 03)	0.9229 (1.30*E* − 03)
	Specificity	0.9862 (2.26*E* − 04)	0.9903 (6.36*E* − 05)	0.9837 (1.40*E* − 03)
	Accuracy	0.9861 (2.24*E* − 04)	0.9901 (6.50*E* − 05)	0.9835 (1.41*E* − 03)
meta-MCP	Sensitivity	0.9651 (1.90*E* − 03)	**0.9362** (**3.90E** − **02**)	0.9205 (2.70*E* − 03)
	Specificity	0.9884 (4.18*E* − 05)	0.9840 (2.02*E* − 05)	0.9846 (1.15*E* − 02)
	Accuracy	0.9883 (4.16*E* − 05)	0.9838 (2.05*E* − 05)	0.9840 (1.13*E* − 02)
meta-SCAD	Sensitivity	**0.9738** (**1.60E** − **03**)	0.9306 (2.07*E* − 03)	0.9392 (2.50*E* − 03)
	Specificity	0.9903 (1.44*E* − 05)	0.9853 (1.20*E* − 04)	0.9505 (4.20*E* − 03)
	Accuracy	0.9903 (1.42*E* − 05)	0.9850 (1.44*E* − 05)	0.9504 (4.10*E* − 03)
meta-LASSO	Sensitivity	0.9065 (8.42*E* − 02)	0.9217 (6.60*E* − 02)	**0.9425** (**6.48E** − **02**)
	Specificity	0.9710 (2.72*E* − 03)	0.9869 (3.07*E* − 03)	0.9940 (1.71*E* − 03)
	Accuracy	0.9708 (2.79*E* − 03)	0.9866 (3.02*E* − 03)	0.9935 (1.69*E* − 03)
composite MCP	Sensitivity	0.8454 (1.46*E* − 01)	0.5428 (1.23*E* − 01)	0.3167 (7.60*E* − 02)
	Specificity	0.9988 (6.02*E* − 04)	0.9992 (4.85*E* − 04)	0.9984 (7.02*E* − 04)
	Accuracy	0.9985 (6.12*E* − 04)	0.9969 (1.02*E* − 03)	0.9922 (1.18*E* − 03)
group Bridge	Sensitivity	0.8734 (7.77*E* − 02)	0.6856 (1.11*E* − 01)	0.2842 (6.27*E* − 02)
	Specificity	0.9997 (2.84*E* − 04)	0.9999 (1.05*E* − 04)	0.9999 (3.49*E* − 05)
	Accuracy	0.9994 (3.42*E* − 04)	0.9983 (6.08*E* − 04)	0.9934 (6.74*E* − 04)
group exponential LASSO	Sensitivity	0.8809 (8.70*E* − 02)	0.7315 (1.67*E* − 01)	0.4661 (2.29*E* − 01)
	Specificity	0.9984 (1.10*E* − 03)	0.9981 (9.33*E* − 04)	0.9976 (1.33*E* − 03)
	Accuracy	0.9981 (1.09*E* − 03)	0.9967 (8.01*E* − 04)	0.9928 (1.37*E* − 03)

### Real-Data analysis

In this section, we apply our methods (meta-Half, meta-MCP and meta-SCAD) to three publicly available lung cancer gene expression datasets, and compare our three methods with other four methods including meta-LASSO, composite MCP, group Bridge and group exponential LASSO.

#### Lung cancer datasets

The three publicly available lung cancer microarray datasets come from disparate platforms and can be download from GEO (https://www.ncbi.nlm.nih.gov/gds/). The three datasets are described as follows:

GSE10072 dataset. The dataset is gene expression signature of cigarette smoking, it contains 107 final expression samples from 58 tumors and 49 non-tumor tissues from 20 never smokers, 26 former smokers, and 28 current smokers, each sample has 22283 genes. The original gene expression data is provided by Landi *et al*.^54^.

GSE19188 dataset. The dataset is expression data for early stage non-small-cell lung cancer (NSCLC), it contains 156 samples from 91 tumor tissues and 65 adjacent normal lung tissue samples, each sample has 54675 genes. The more information can be found in Hou *et al*.^55^.

GSE19804 dataset. The dataset is non-smoking female lung cancer in Taiwan, it contains 120 samples from 60 tumors and 60 normal tumor tissues, each sample has 54675 genes. The more information can be found in Lu *et al*.^56^.

Each dataset is divided into two parts, about 70 percent of the datasets as training samples and the other 30 percent as testing samples. Table 2 lists the details of the three datasets.

**Table 2:** The description of three publicly available lung cancer gene expression datasets.

Dataset	No. of Probs	Classes (Class 0/Class 1)	No. of samples (Class 0/Class 1)	Affymetrix Platform
GSE10072	22284	Normal/ Lung Cancer	107 (49/58)	U133A
GSE19188	54675	Normal/ Lung Cancer	156 (65/91)	U133 Plus 2.0
GSE19804	54676	Normal/ Lung Cancer	120 (60/60)	U133 Plus 2.0

The original Affymetrix data was first normalized and log-transformed by a robust multi-array average (RMA) method^57^. After that, downloading and installing the appropriate custom chip definition files (CDFs) packages according to the type of microarray platform. The CDF package is necessary for probe annotation for Affymetrix data. The probes of the normalized data can be successfully mapped to Entrez Gene IDs by annotation packages in Bioconductor^58^. If multiple probes match a single Entrez ID, we calculated the median of values of those probes as the expression value for this gene.

We extract common genes from the three gene expression datasets as the merged set of genes. There are 13515 common genes in three datasets and our analysis is based on those 13515 genes. We use a random partition in three lung cancer datasets, and apply aforementioned seven methods to select important genes, with the optimal tuning parameters chosen by the BIC as discussed above. We repeat this procedure 30 times and report the average measurement and standard error.

#### Evaluating the classification performance

**Figure 2:**
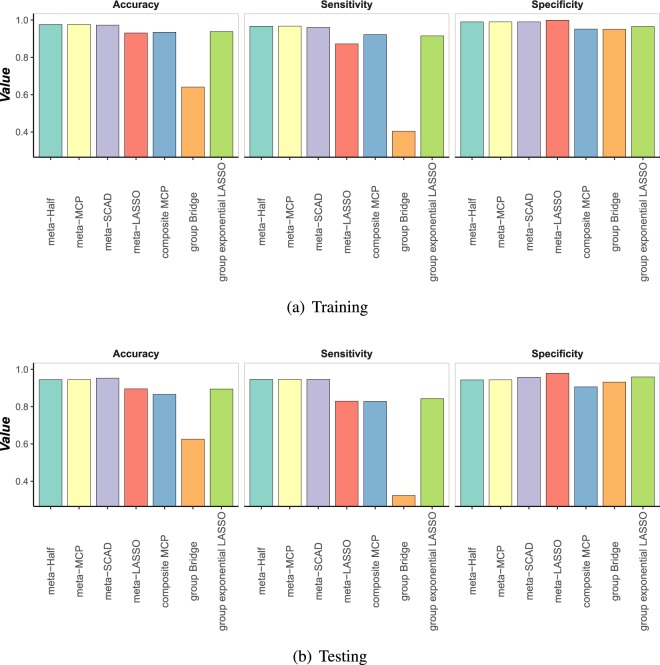
Training and testing prediction performance of different methods on lung cancer datasets. (**a**) Training. (**b**) Testing.

 Table 3 demonstrates the prediction performance of the seven methods in three lung cancer datasets. The sensitivity, specificity and accuracy of training and testing predictions for all seven methods are shown in Fig. 2.

**Table 3:** Performance comparisons of different methods in three lung cancer datasets. Presented values are the average (standard error).

Methods	Training data			Testing data		
	Accuracy	Sensitivity	Specificity	Accuracy	Sensitivity	Specificity
meta-Half	0.9766 (2.69E-02)	0.9673 (9.10E-03)	0.9903 (1.54E-05)	0.9449 (2.16E-02)	0.9464 (7.93E-03)	0.9437 (1.66E-05)
meta-MCP	**0.9768** (5.37E-04)	**0.9677** (1.70E-03)	0.9903 (8.56E-05)	0.9452 (1.99E-03)	**0.9466** (6.94E-03)	0.9439 (1.35E-04)
meta-SCAD	0.9727 (3.13E-03)	0.9608 (1.77E-03)	0.9903 (2.19E-02)	**0.9528** (4.64E-03)	0.9464 (4.80E-03)	0.9577 (2.50E-02)
meta-LASSO	0.9309 (1.01E-02)	0.8722 (1.87E-02)	**0.9994** (2.15E-03)	0.8953 (2.11E-02)	0.8291 (3.58E-02)	**0.9792** (1.25E-02)
composite MCP	0.9353 (1.45E-02)	0.9221 (2.05E-02)	0.9519 (1.85E-02)	0.8656 (1.94E-02)	0.8283 (2.23E-02)	0.9060 (2.85E-02)
group Bridge	0.6410 (1.88E-02)	0.4039 (2.59E-02)	0.9508 (2.23E-02)	0.6255 (2.06E-02)	0.3240 (3.15E-02)	0.9317 (2.98E-02)
group exponential Lasso	0.9385 (9.78E-03)	0.9155 (1.64E-02)	0.9655 (1.48E-02)	0.8942 (1.85E-02)	0.8432 (2.47E-02)	0.9589 (2.05E-02)

As shown in Table 3 and Fig. 2, for the training dataset and testing dataset, the sensitivity and accuracy of meta-Half, meta-MCP, meta-SCAD are consistently higher than the other four methods, and the specificity of all methods are similar. This result shows that our three methods are more effectively distinguish whether an individual is a disease patient compared to the other four methods. Therefore, our three methods have superior performance than the other four methods in the prediction and diagnosis of diseases.

#### Analysis of the selected genes

 Table 4 gives the names of genes selected in each dataset. We focus on the gene WIF1 which is bolded in the Table 4. WIF1, a secreted Wnt antagonist, is a downstream gene of the Wnt/*β*-catenin pathway, which exerts inhibition through direct binding to Wnt proteins^59^. WIF1 was found to be silenced by methylation in various human carcinomas including lung^60^, oral^61^, nasopharyngeal^62^, esophageal^63^, breast^64^ and colon cancer^65^ etc.

**Table 4:** Gene selections of seven methods in three lung cancer datasets.

	GSE10072			GSE19188			GSE19804		
meta-Half	CXCL13	MMP12	COL11A1	CXCL13	MMP12	COL11A1	CXCL13	SPINK1	AGER
	TOX3	SPINK1	FCN3	TOX3	SPINK1	FCN3	SPP1	COL10A1	GPM6A
	SPP1	COL10A1	**WIF1**	SPP1	COL10A1	**WIF1**	MMP12	TOX3	
	GPM6A	AGER		GPM6A	AGER				
meta-MCP	CXCL13	SPP1	COL11A1	CXCL13	SPP1	COL11A1	CXCL13	SPP1	
	AGER	MMP12	FCN3	AGER	MMP12	FCN3	AGER	MMP12	
	PPAP2C	COL10A1	**WIF1**	PPAP2C	COL10A1	**WIF1**	PPAP2C	COL10A1	
	GPM6A	TOX3		GPM6A	TOX3		GPM6A	TOX3	
	TOP2A	TMEM100		TOP2A	TMEM100		TOP2A	TMEM100	
meta-SCAD	CXCL13	MMP12	COL11A1	CXCL13	MMP12	COL11A1	CXCL13	MMP12	COL11A1
	CYP4B1	SPINK1	FCN3	CYP4B1	SPINK1	FCN3	CYP4B1	SPINK1	FCN3
	SPP1	AGER	**WIF1**	SPP1	AGER	**WIF1**	SPP1	AGER	
meta-LASSO	PPBP	SFTPC	SPP1	PPBP	SFTPC	SPP1	PPBP	SFTPC	SPP1
	CLDN10	AKR1B10	SFTPD	CLDN10	AKR1B10	SFTPD	CLDN10	AKR1B10	SFTPD
	UPK3B	APOLD1	XIST	UPK3B	APOLD1	XIST	UPK3B	APOLD1	XIST
	SPINK1	COL10A1	**WIF1**	SPINK1	COL10A1	**WIF1**	SPINK1	COL10A1	**WIF1**
	HLA-DQA1 /// LOC100509457			HLA-DQA1 /// LOC100509457			HLA-DQA1 /// LOC100509457		
composite MCP	SOSTDC1	COL11A1		SYNE1			SOSTDC1	COL11A1	
group Bridge	P2RY14			P2RY14	ATP1A2		P2RY14		
group	GDF10	FABP4	COL11A1	GDF10	FABP4	COL11A1	GDF10	FABP4	COL11A1
exponential									
LASSO									

As shown in Table 4, our three methods (meta-Half, meta-MCP and meta-SCAD) all select gene WIF1 on both datasets GSE10072 and GSE19188, but not select gene WIF1 on dataset GSE19804. As we known that GSE10072 dataset is gene expression signature of cigarette smoking, GSE19188 dataset is expression data for early stage non-small-cell lung cancer (NSCLC) and GSE19804 dataset is non-smoking female lung cancer in Taiwan. Huang *et al*. showed that WIF1 is significantly associated with the smoking behavior in NSCLC patients^66^. It shows that our three methods can more realistically identify the important biomarkers from different datasets which have heterogeneity. The meta-LASSO selects gene WIF1 in all three lung cancer datasets, and the genes selected by meta-LASSO in the three lung cancer datasets are the same. The other three methods (composite MCP, group Bridge and group exponential LASSO) cannot select gene WIF1 in all three lung cancer datasets. Therefore, our three methods are superior to the other four methods when applied in the heterogeneity datasets.

**Figure 3:**
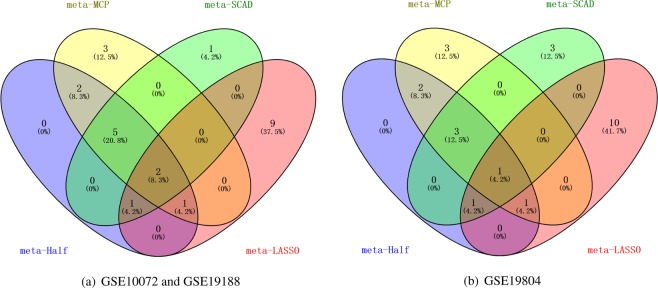
Overlap of commonly selected genes across the different methods in lung cancer datasets. (**a**) GSE10072 and GSE19188. (**b**) GSE19804.

The number of genes selected by meta-Half, meta-MCP, meta-SCAD and meta-LASSO are 11, 13, 9 and 13 respectively. Figure 3 shows the overlap of commonly selected genes across the four different methods (meta-Half, meta-MCP, meta-SCAD, meta-LASSO) in three lung cancer datasets. The other three methods (composite MCP, group Bridge and group exponential LASSO) select fewer genes, so we don’t show the genes they selected in Fig. 3. As shown in Fig. 3(a), for the datasets GSE10072 and GSE19188, seven common genes are selected by meta-Half, meta-MCP and meta-SCAD, which are CXCL13, COL11A1, SPP1, MMP12, AGER, WIF1 and FCN3. Two common genes are selected by meta-Half, meta-MCP, meta-SCAD and meta-LASSO, which are SPP1 and WIF1. Figure 3(b) shows that for dataset GSE19804, four common genes selected by meta-Half, meta-MCP and meta-SCAD are CXCL13, SPP1, MMP12 and AGER. One common genes are selected by meta-Half, meta-MCP, meta-SCAD and meta-LASSO, which is SPP1. More unique non-overlapping sets of genes are selected by our three methods and meta-LASSO. In addition, some of the aforementioned genes have been reported in the literature. COL11A1 is collagen type XI alpha 1 chain. The over-expression of COL11A1 reportedly correlates with lymph node metastasis and poor prognosis in non-small cell lung cancer and ovarian cancer^67^. Zhang *et al*. suggest that SPP1 and AGER are risk factors for lung adenocarcinoma, and these two genes may be utilized in the prognostic evaluation of patients with lung adenocarcinoma^68^. The advanced glycosylation end-product specific receptor (AGER) belongs to the immunoglobulin superfamily, whose abnormal expression has been detected in lung cancer^69^. MMP12 is matrix metallopeptidase 12 and may play a role in aneurysm formation and mutations in this gene are associated with lung function and chronic obstructive pulmonary disease (COPD)^70^. WIF1 was found to be silenced by methylation in lung^60^. Lea *et al*. shows that the Ficolin-3, encoded by the FCN3 gene and expressed in the lung and liver, is a recognition molecule in the lectin pathway of the complement system^71^. The aforementioned genes CXCL13, MMP12, AGER and FCN3 are only selected by our three methods, and the gene COL11A1 is selected by our three methods and group exponential LASSO.

**Figure 4:**
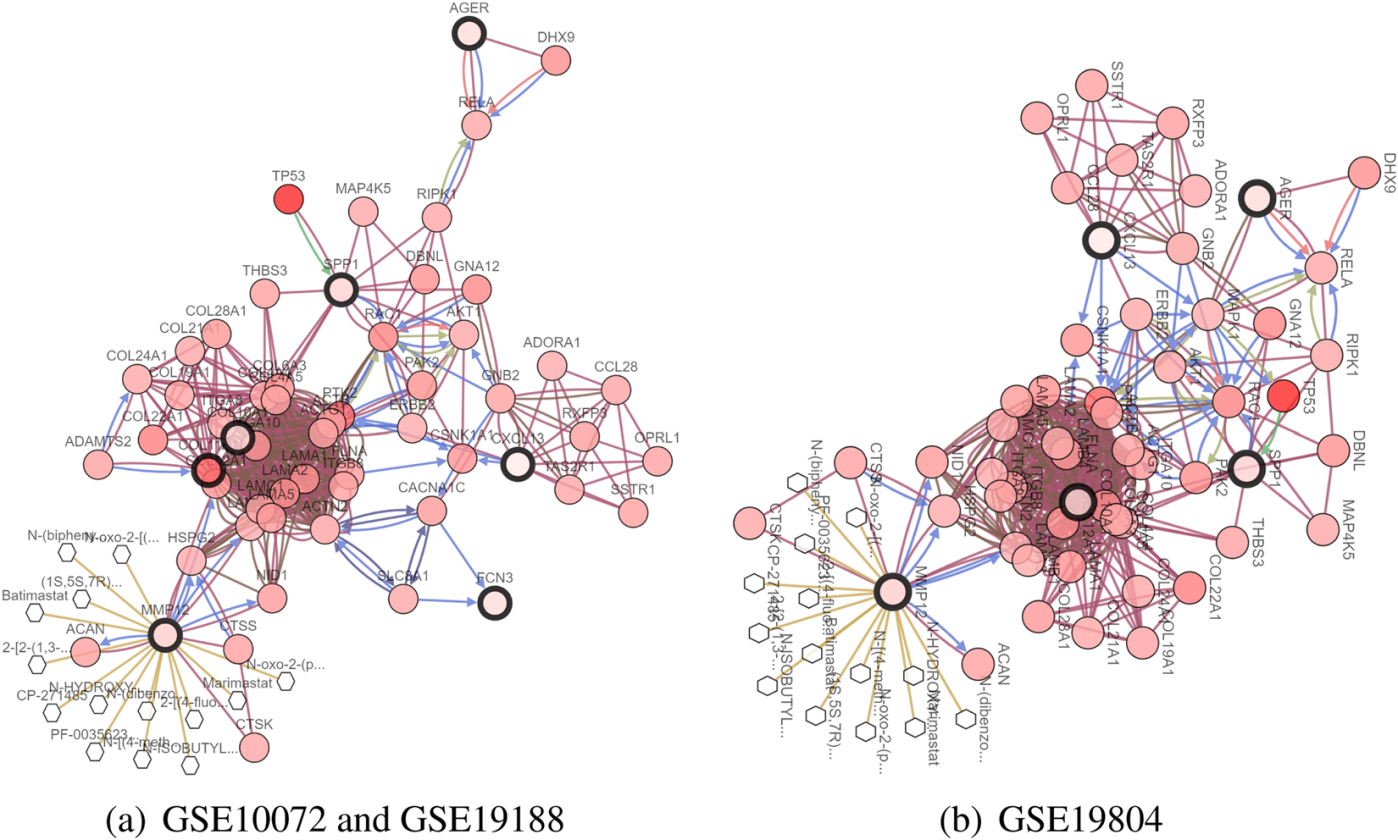
Network view of the genes selected from meta-Half in lung cancer datasets. The genes corresponding to the selected variables are highlighted by a thicker black outline. The rest of the nodes correspond to the genes that are frequently altered and are known to interact with the highlighted genes (based on publicly available interaction data). The nodes are gradient color-coded according to the alteration frequency based on microarray data derived from the TCGA lung cancer dataset via cBioPortal. (**a**) GSE10072 and GSE19188. (**b**) GSE19804.

**Figure 5:**
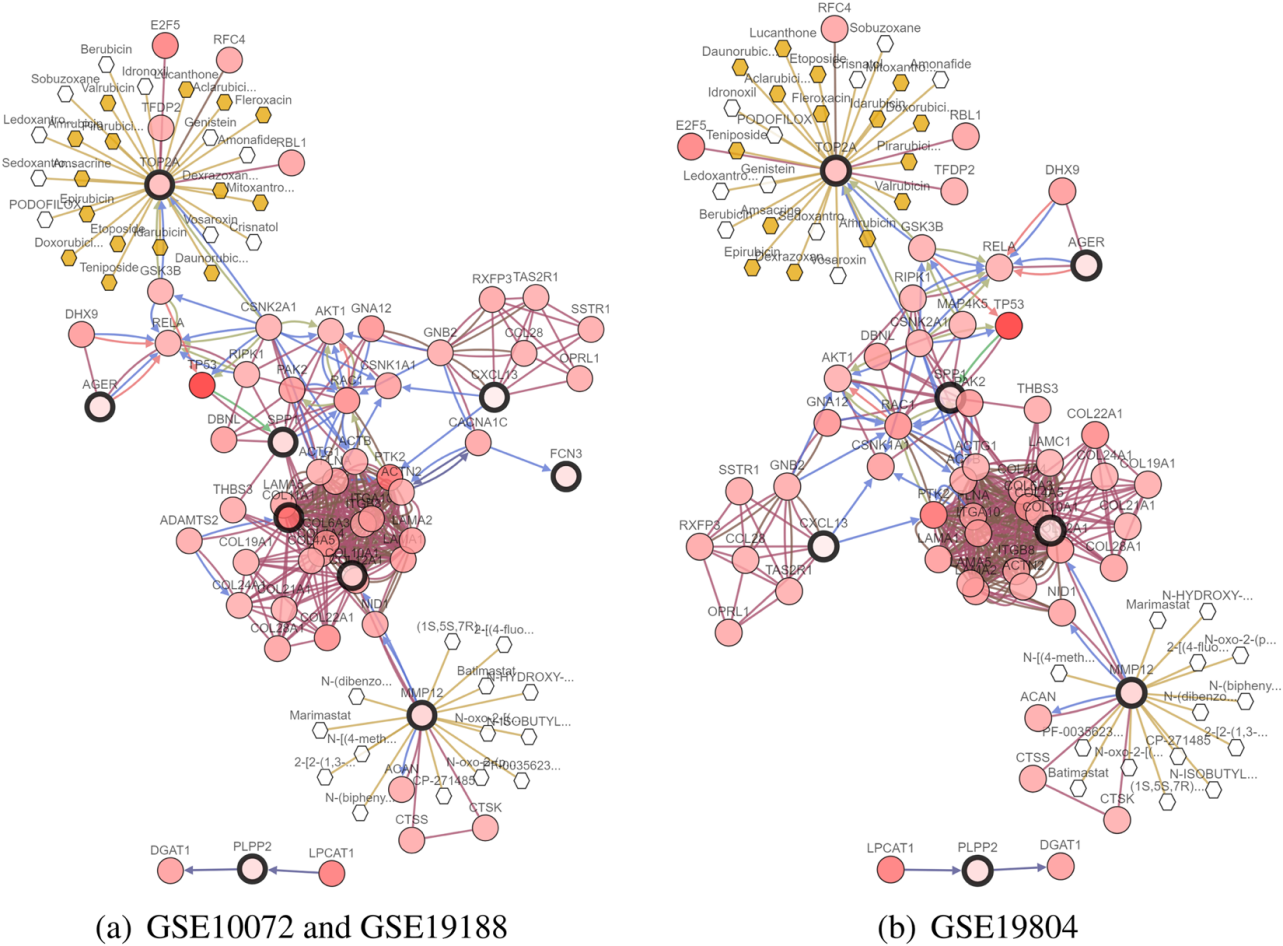
Network view of the genes selected from meta-MCP in lung cancer datasets. (**a**) GSE10072 and GSE19188. (**b**) GSE19804.

**Figure 6:**
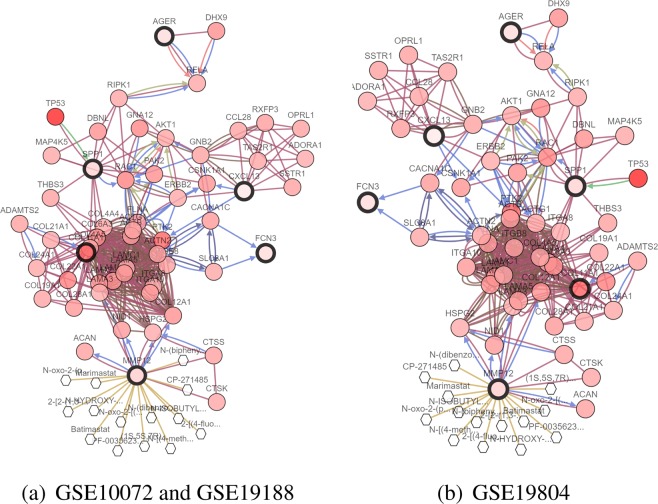
Network view of the genes selected from meta-SCAD in lung cancer datasets. (**a**) GSE10072 and GSE19188. (**b**) GSE19804.

To make it easier to demonstrate the interplay between the selected genes from the different methods, we construct a network of interactions among the genes using the cBioPortal^72,73^. Figures 4, 5 and 6 show the interactive network of the genes selected by our three methods in three lung cancer datasets. Most of the genes selected by our three methods are linked to the frequently altered neighbor genes from the TCGA lung adenocarcinoma dataset. The expression of SPP1 is controlled by TP53. TP53 is tumor protein p53, this gene encodes a tumor suppressor protein containing transcriptional activation, DNA binding, and oligomerization domains. Mutations in this gene are associated with a variety of human cancers^74^. MMP12 and TOP2A are targeted by certain cancer drugs, and are only selected by our three methods.

In this part, we analyze the genes selected by the four methods (meta-Half, meta-MCP, meta-SCAD and meta-LASSO) in three lung cancer datasets. According to the network of interactions between genes, among the genes selected by our three methods, we find that some genes are connected to other frequently altered genes in publicly available datasets, and some genes are targeted by certain cancer drugs. Some functions may also need to be verified in the future. Results demonstrate that our three methods have good performance in the high-dimensionality gene expression data with heterogeneity.

## Conclusion

With the rapid development of biotechnology and its wide applications, a large number of publicly available gene expression datasets have been produced. However, due to the gene expression datasets have the characteristics of small sample size, high dimensionality and high noise, the application of biostatistics and machine learning methods to analyze gene expression data is a challenging task, such as the low reproducibility of important biomarkers in different studies. The low reproducibility of important biomarkers is mainly caused by the heterogeneity of the different datasets. These problems reveal the complexity of gene expression data and significantly obstruct biotechnology in clinical applications. Meta-analysis is an effective approach to deal with these problems. It plays an important role in summarizing and synthesizing scientific evidence from multiple studies, and provides a more comprehensive understanding of the biological systems, but the current methods have some limitations. The nonconvex regularization method is an effective approach for variable selection developed in recent years. In this paper, we combine the advantages of meta-analysis and the nonconvex regularization method, and propose three novel methods, dubbed as meta-Half, meta-MCP and meta-SCAD, respectively. Through the hierarchical decomposition of coefficients, our methods not only consider the data heterogeneity to maintain the flexibility in selecting variables on different datasets, but also consider the correlation between multiple datasets to improve the ability of identifying important biomarkers. We give the efficient algorithms which apply the nonconvex iterative thresholding algorithms based on approximate message passing (Half-AMP, MCP-AMP and SCAD-AMP) to solve our models and study the theoretical property of meta-Half. The theoretical property analysis of MCP-AMP and SCAD-AMP are the future work. We prove meta-Half possesses sparsity, unbiasedness and oracle properties. Furthermore, we apply our methods to the simulation data and three publicly available lung cancer gene expression datasets, and compare the performance of our methods with other four methods, which are meta-LASSO, composite MCP, group Bridge and group exponential LASSO. Simulation studies demonstrate our methods have the superior performance when data heterogeneity is strong. In the three publicly available lung cancer gene expression datasets, the analysis results show that our three methods have good performance in the gene expression data of small sample size and high dimensionality from different sources (heterogeneity), and the selected important biomarkers have clinical significance. Our methods can also be extended to other areas where datasets are heterogeneous.

## Supplementary information

LaTeX Supplementary File.
