# Supplementary material for: Meta-Analysis Based on Nonconvex Regularization
Source: Sci Rep. 2020 Apr 1;10:5755. doi: 10.1038/s41598-020-62473-2 (PMC7113298; doi:10.1038/s41598-020-62473-2)
Supplement: Supplementary file 1 — LaTeX Supplementary File. [file 41598_2020_62473_MOESM1_ESM.pdf]

Supplementary Material: Meta-Analysis Based on Nonconvex Regularization

Hui Zhang[1], Shou-Jiang Li[1], Hai Zhang[1,2], Zi-Yi Yang[1], Yan-Qiong Ren[1], Liang-Yong Xia[1], and Yong Liang[1,*]

[1]Faculty of Information Technology & State Key Laboratory of Quality Research in Chinese Medicines, Macau University of Science and Technology, Taipa , 999078, Macau

[2]School of Mathematics, Northwest University, 710127, Xi'an People's Republic of China

[*]Correspondence: yliang@must.edu.mo

Appendix: Proofs of Lemmas and Theorems

Proof of Lemma 1

Let $Q_1(\lambda_h, \lambda_\xi, \boldsymbol{\beta}_0, \boldsymbol{h}, \boldsymbol{\xi})$ denote the objective function in (19) and $Q_2(\lambda, \boldsymbol{\beta}_0, \boldsymbol{h}, \boldsymbol{\xi})$ denote the objective function in (20). suppose $(\tilde{\boldsymbol{\beta}}_0, \tilde{\boldsymbol{h}}, \tilde{\boldsymbol{\xi}})$ is the local maximizer of $Q_1(\lambda_h, \lambda_\xi, \boldsymbol{\beta}_0, \boldsymbol{h}, \boldsymbol{\xi})$, we would like to show that $(\hat{\boldsymbol{\beta}}_0, \hat{\boldsymbol{h}}, \hat{\boldsymbol{\xi}}) = (\tilde{\boldsymbol{\beta}}_0, \lambda_h^2 \tilde{\boldsymbol{h}}, \frac{1}{\lambda_h^2}\tilde{\boldsymbol{\xi}})$ is a local maximizer of $Q_2(\lambda, \boldsymbol{\beta}_0, \boldsymbol{h}, \boldsymbol{\xi})$.

Since $(\tilde{\boldsymbol{\beta}}_0, \tilde{\boldsymbol{h}}, \tilde{\boldsymbol{\xi}})$ is the local maximizer of (19), there exist $\delta > 0$, such that for any $(\boldsymbol{\beta}_0, \boldsymbol{h}, \boldsymbol{\xi})$ satisfying $\|\boldsymbol{\beta}_0 - \tilde{\boldsymbol{\beta}}_0\|_1 + \|\boldsymbol{h} - \tilde{\boldsymbol{h}}\|_1 + \|\boldsymbol{\xi} - \tilde{\boldsymbol{\xi}}\|_1 \le \delta$, $Q_1(\lambda_h, \lambda_\xi, \boldsymbol{\beta}_0, \boldsymbol{h}, \boldsymbol{\xi}) \le Q_1(\lambda_h, \lambda_\xi, \tilde{\boldsymbol{\beta}}_0, \tilde{\boldsymbol{h}}, \tilde{\boldsymbol{\xi}})$. Choose δ' such that $\frac{\delta'}{\min(\lambda_h^2, \frac{1}{\lambda_h^2})} \le \delta$, then for any $(\boldsymbol{\beta}_0', \boldsymbol{h}', \boldsymbol{\xi}')$ satisfying $\|\boldsymbol{\beta}_0' - \hat{\boldsymbol{\beta}}_0\|_1 + \|\boldsymbol{h}' - \hat{\boldsymbol{h}}\|_1 + \|\boldsymbol{\xi}' - \hat{\boldsymbol{\xi}}\|_1 \le \delta'$, it holds that

$$
\begin{aligned}
\|\boldsymbol{\beta}_0' - \tilde{\boldsymbol{\beta}}_0\|_1 + \|\frac{\boldsymbol{h}'}{\lambda_h^2} - \tilde{\boldsymbol{h}}\|_1 + \|\lambda_h^2 \boldsymbol{\xi}' - \tilde{\boldsymbol{\xi}}\|_1 &\le \frac{\|\boldsymbol{\beta}_0' - \tilde{\boldsymbol{\beta}}_0\|_1 + \lambda_h^2 \|\frac{\boldsymbol{h}'}{\lambda_h^2} - \tilde{\boldsymbol{h}}\|_1 + \frac{1}{\lambda_h^2}\|\lambda_h^2 \boldsymbol{\xi}' - \tilde{\boldsymbol{\xi}}\|_1}{\min(\lambda_h^2, \frac{1}{\lambda_h^2})} \\
&= \frac{\|\boldsymbol{\beta}_0' - \hat{\boldsymbol{\beta}}_0\|_1 + \|\boldsymbol{h}' - \hat{\boldsymbol{h}}\|_1 + \|\boldsymbol{\xi}' - \hat{\boldsymbol{\xi}}\|_1}{\min(\lambda_h^2, \frac{1}{\lambda_h^2})} \\
&< \delta.
\end{aligned}
$$

Hence,

$$
\begin{aligned}
Q_2(\lambda, \boldsymbol{\beta}_0', \boldsymbol{h}', \boldsymbol{\xi}') &= Q_1(\lambda_h, \lambda_\xi, \boldsymbol{\beta}_0', \frac{\boldsymbol{h}'}{\lambda_h^2}, \lambda_h^2 \boldsymbol{\xi}') \\
&\le Q_1(\lambda_h, \lambda_\xi, \tilde{\boldsymbol{\beta}}_0, \tilde{\boldsymbol{h}}, \tilde{\boldsymbol{\xi}}) \\
&= Q_2(\lambda, \hat{\boldsymbol{\beta}}_0, \hat{\boldsymbol{h}}, \hat{\boldsymbol{\xi}}).
\end{aligned}
$$

Therefore, $(\hat{\boldsymbol{\beta}}_0, \hat{\boldsymbol{h}}, \hat{\boldsymbol{\xi}}) = (\tilde{\boldsymbol{\beta}}_0, \lambda_h^2 \tilde{\boldsymbol{h}}, \frac{1}{\lambda_h^2}\tilde{\boldsymbol{\xi}})$ is a local maximizer of $Q_2(\lambda, \boldsymbol{\beta}_0, \boldsymbol{h}, \boldsymbol{\xi})$. Similarly, we can show the reverse.

Proof of Lemma 2

(a) If $\hat{h}_j = 0$, then $\hat{\boldsymbol{\beta}}_j = \boldsymbol{0}$ is obvious.

(b) We prove (b) by contradiction. Suppose there exists j' such that $\hat{h}_j \neq 0$ and $\hat{h}_j \neq \lambda \|\hat{\boldsymbol{\beta}}_j\|_{1/2}^{1/2}$. Let $\frac{\lambda \|\hat{\boldsymbol{\beta}}_j\|_{1/2}^{1/2}}{\hat{h}_j} = C$, then $\hat{\boldsymbol{\xi}}_j = \frac{C\hat{\boldsymbol{\beta}}_j}{\lambda \|\hat{\boldsymbol{\beta}}_j\|_{1/2}^{1/2}}$.

We first consider the case of $C > 1$. Let $\tilde{h}_j = \hat{h}_j$, $\tilde{\boldsymbol{\xi}}_j = \hat{\boldsymbol{\xi}}_j$ for $j \neq j'$, and $\tilde{h}_{j'} = \delta' \hat{h}_{j'}$, $\tilde{\boldsymbol{\xi}}_{j'} = \frac{1}{\delta'}\hat{\boldsymbol{\xi}}_{j'}$, where δ' satisfies $1 < \delta' < C$ and is very close to 1 such that $|\tilde{h}_{j'} - \hat{h}_{j'}| + \|\tilde{\boldsymbol{\xi}}_{j'} - \hat{\boldsymbol{\xi}}_{j'}\|_1 < \delta$

for some $\delta > 0$. Then, we have

$$
\begin{aligned}
Q_2(\lambda, \hat{\boldsymbol{\beta}}_0, \tilde{\boldsymbol{h}}, \tilde{\boldsymbol{\xi}}) - Q_2(\lambda, \hat{\boldsymbol{\beta}}_0, \hat{\boldsymbol{h}}, \hat{\boldsymbol{\xi}}) &= -\sqrt{\delta'}|\hat{h}_{j'}|^{\frac{1}{2}} - \frac{1}{\sqrt{\delta'}}\lambda \sum_{m=1}^{M} |\hat{\xi}_{mj'}|^{\frac{1}{2}} + |\hat{h}_{j'}|^{\frac{1}{2}} + \lambda \sum_{m=1}^{M} |\hat{\xi}_{mj'}|^{\frac{1}{2}} \\
&= \frac{1-\sqrt{\delta'}}{\sqrt{C}}\sqrt{\lambda\|\hat{\boldsymbol{\beta}}_j\|_{1/2}^{1/2}} + (1 - \frac{1}{\sqrt{\delta'}})\sqrt{C}\sqrt{\lambda\|\hat{\boldsymbol{\beta}}_j\|_{1/2}^{1/2}} \\
&= (\frac{1}{\sqrt{C}} - \sqrt{\frac{\delta'}{C}} + \sqrt{C} - \sqrt{\frac{C}{\delta'}})\sqrt{\lambda\|\hat{\boldsymbol{\beta}}_j\|_{1/2}^{1/2}} \\
&= \frac{1}{\sqrt{C}}(\sqrt{\delta'} - 1)(\frac{C}{\sqrt{\delta'}} - 1)\sqrt{\lambda\|\hat{\boldsymbol{\beta}}_j\|_{1/2}^{1/2}} \\
&> 0.
\end{aligned}
$$

Therefore, for any $\delta > 0$, we can find $\tilde{\boldsymbol{h}}, \tilde{\boldsymbol{\xi}}$ such that $\|\tilde{\boldsymbol{h}} - \hat{\boldsymbol{h}}\|_1 + \|\tilde{\boldsymbol{\xi}} - \hat{\boldsymbol{\xi}}\|_1 < \delta$ and $Q_2(\lambda, \hat{\boldsymbol{\beta}}_0, \tilde{\boldsymbol{h}}, \tilde{\boldsymbol{\xi}}) > Q_2(\lambda, \hat{\boldsymbol{\beta}}_0, \hat{\boldsymbol{h}}, \hat{\boldsymbol{\xi}})$. These contradict the fact that $(\hat{\boldsymbol{h}}, \hat{\boldsymbol{\xi}})$ is a local maximizer of (20).

For the case of $0 < C < 1$, there are similar results, we omit here.

Hence, we obtain the result that if $\hat{h}_j \neq 0$, then $\hat{\boldsymbol{\beta}}_j \neq \mathbf{0}$ and $\hat{h}_j = \lambda\|\hat{\boldsymbol{\beta}}_j\|_{1/2}^{1/2}$, $\hat{\boldsymbol{\xi}}_j = \frac{\hat{\boldsymbol{\beta}}_j}{\lambda\|\hat{\boldsymbol{\beta}}_j\|_{1/2}^{1/2}}$

Proof of Theorem 1

Let Q_3 be the corresponding criterion in equation (21). Suppose $(\hat{\boldsymbol{h}}, \hat{\boldsymbol{\xi}})$ is a local maximizer of $Q_2(\lambda, \boldsymbol{h}, \boldsymbol{\xi})$. We first show that $\hat{\boldsymbol{\beta}}$, where $\hat{\beta}_{mj} = \hat{h}_j \hat{\xi}_{mj}$, is a local maximizer of $Q_3(\lambda, \boldsymbol{\beta})$, i.e. there exists a δ' such that if $\|\triangle\boldsymbol{\beta}\|_1 < \delta'$ then $Q_3(\lambda, \hat{\boldsymbol{\beta}} + \triangle\boldsymbol{\beta}) \leq Q_3(\lambda, \hat{\boldsymbol{\beta}})$.

We denote $\triangle\boldsymbol{\beta} = \triangle\boldsymbol{\beta}^{(1)} + \triangle\boldsymbol{\beta}^{(2)}$, where $\triangle\boldsymbol{\beta}_j^{(1)} = \mathbf{0}$ if $\|\hat{\boldsymbol{\beta}}_j\|_1 = 0$ and $\triangle\boldsymbol{\beta}_j^{(2)} = \mathbf{0}$ if $\|\hat{\boldsymbol{\beta}}_j\|_1 \neq 0$, we have $\|\triangle\boldsymbol{\beta}\|_1 = \|\triangle\boldsymbol{\beta}^{(1)}\|_1 + \|\triangle\boldsymbol{\beta}^{(2)}\|_1$.

Now we show that there exists $\delta' > 0$, such that for any $\|\triangle\boldsymbol{\beta}\|_1 < \delta'$, $Q_3(\lambda, \hat{\boldsymbol{\beta}} + \triangle\boldsymbol{\beta}) \leq Q_3(\lambda, \hat{\boldsymbol{\beta}})$. By Lemma 2, for $j = 1, 2, \cdots, p$, we have if $\hat{h}_j \neq 0$, then $\hat{\boldsymbol{\beta}}_j \neq \mathbf{0}$, $\hat{h}_j = \lambda\|\hat{\boldsymbol{\beta}}_j\|_{1/2}^{1/2}$, $\hat{\boldsymbol{\xi}}_j = \frac{\hat{\boldsymbol{\beta}}_j}{\lambda\|\hat{\boldsymbol{\beta}}_j\|_{1/2}^{1/2}}$ and if $\hat{h}_j = 0$, then $\hat{\boldsymbol{\xi}}_j = \mathbf{0}$. Furthermore, if $\hat{h}_j \neq 0$, let

$$\hat{h}'_j = \lambda\|\hat{\boldsymbol{\beta}}_j + \triangle\boldsymbol{\beta}_j^{(1)}\|_{1/2}^{1/2},$$

$$\hat{\boldsymbol{\xi}}'_j = \frac{\hat{\boldsymbol{\beta}}_j + \triangle\boldsymbol{\beta}_j^{(1)}}{\lambda\|\hat{\boldsymbol{\beta}}_j + \triangle\boldsymbol{\beta}_j^{(1)}\|_{1/2}^{1/2}},$$

and if $\hat{h}_j = 0$, let $\hat{h}'_j = 0$, $\hat{\boldsymbol{\xi}}'_j = 0$. then we have $Q_2(\lambda, \hat{\boldsymbol{h}}, \hat{\boldsymbol{\xi}}) = Q_3(\lambda, \hat{\boldsymbol{\beta}})$ and $Q_2(\lambda, \hat{\boldsymbol{h}}', \hat{\boldsymbol{\xi}}') = Q_3(\lambda, \hat{\boldsymbol{\beta}} + \triangle\boldsymbol{\beta}^{(1)})$. Therefore, we only need to show that $Q_2(\lambda, \hat{\boldsymbol{h}}', \hat{\boldsymbol{\xi}}') \leq Q_2(\lambda, \hat{\boldsymbol{h}}, \hat{\boldsymbol{\xi}})$. Since $(\hat{\boldsymbol{h}}, \hat{\boldsymbol{\xi}})$ is a local maximizer of $Q_2(\lambda, \boldsymbol{h}, \boldsymbol{\xi})$, there exists $\delta > 0$, for any $\hat{\boldsymbol{h}}', \hat{\boldsymbol{\xi}}'$ satisfying $\|\hat{\boldsymbol{h}}' - \hat{\boldsymbol{h}}\|_1 + \|\hat{\boldsymbol{\xi}}' - \hat{\boldsymbol{\xi}}\|_1 < \delta$, we have $Q_2(\lambda, \hat{\boldsymbol{h}}', \hat{\boldsymbol{\xi}}') \leq Q_2(\lambda, \hat{\boldsymbol{h}}, \hat{\boldsymbol{\xi}})$. Note that

$$
\begin{aligned}
|\hat{h}'_j - \hat{h}_j| &= |\lambda \|\hat{\boldsymbol{\beta}}_j + \triangle\boldsymbol{\beta}_j^{(1)}\|_{1/2}^{1/2} - \lambda\|\hat{\boldsymbol{\beta}}_j\|_{1/2}^{1/2}| \\
&= |\lambda \sum_{m=1}^{M} \frac{|\hat{\beta}_{mj} + \triangle\beta_{mj}^{(1)}| - |\hat{\beta}_{mj}|}{|\hat{\beta}_{mj} + \triangle\beta_{mj}^{(1)}|^{\frac{1}{2}} - |\hat{\beta}_{mj}|^{\frac{1}{2}}}| \\
&\le |\lambda \sum_{m=1}^{M} \frac{|\triangle\beta_{mj}^{(1)}|}{2\sqrt{|\hat{\beta}_{mj}| - |\triangle\beta_{mj}^{(1)}|}}| \\
&\le \frac{\lambda}{2} |\sum_{m=1}^{M} \frac{|\triangle\beta_{mj}^{(1)}|}{\sqrt{d-\delta'}}| \\
&\le \frac{\lambda}{2} \frac{\|\triangle\hat{\boldsymbol{\beta}}_j^{(1)}\|_1}{\sqrt{d/2}},
\end{aligned}
$$

where $d = \min\{|\hat{\beta}_{mj}| : |\hat{\beta}_{mj}| \neq 0\}$ and $\delta' < \frac{d}{2}$. Meanwhile,

$$
\begin{aligned}
\|\hat{\boldsymbol{\xi}}'_j - \hat{\boldsymbol{\xi}}_j\|_1 &= \| \frac{\hat{\boldsymbol{\beta}}_j + \triangle\boldsymbol{\beta}_j^{(1)}}{\lambda\|\hat{\boldsymbol{\beta}}_j + \triangle\boldsymbol{\beta}_j^{(1)}\|_{1/2}^{1/2}} - \frac{\hat{\boldsymbol{\beta}}_j}{\lambda\|\hat{\boldsymbol{\beta}}_j\|_{1/2}^{1/2}} \|_1 \\
&\le \| \frac{\hat{\boldsymbol{\beta}}_j + \triangle\boldsymbol{\beta}_j^{(1)}}{\lambda\|\hat{\boldsymbol{\beta}}_j + \triangle\boldsymbol{\beta}_j^{(1)}\|_{1/2}^{1/2}} - \frac{\hat{\boldsymbol{\beta}}_j}{\lambda\|\hat{\boldsymbol{\beta}}_j + \triangle\boldsymbol{\beta}_j^{(1)}\|_{1/2}^{1/2}} \|_1 + \\
&\quad \| \frac{\hat{\boldsymbol{\beta}}_j}{\lambda\|\hat{\boldsymbol{\beta}}_j + \triangle\boldsymbol{\beta}_j^{(1)}\|_{1/2}^{1/2}} - \frac{\hat{\boldsymbol{\beta}}_j}{\lambda\|\hat{\boldsymbol{\beta}}_j\|_{1/2}^{1/2}} \|_1 \\
&\le \frac{\|\triangle\boldsymbol{\beta}_j^{(1)}\|_1}{\lambda M\sqrt{d/2}} + \|\hat{\boldsymbol{\beta}}_j\|_1 \frac{|\lambda\|\hat{\boldsymbol{\beta}}_j + \triangle\boldsymbol{\beta}_j^{(1)}\|_{1/2}^{1/2} - \lambda\|\hat{\boldsymbol{\beta}}_j\|_{1/2}^{1/2}|}{\lambda\|\hat{\boldsymbol{\beta}}_j + \triangle\boldsymbol{\beta}_j^{(1)}\|_{1/2}^{1/2} \cdot \lambda\|\hat{\boldsymbol{\beta}}_j\|_{1/2}^{1/2}} \\
&\le \|\triangle\boldsymbol{\beta}_j^{(1)}\|_1 \frac{1}{\lambda M\sqrt{d/2}} + D\frac{\|\triangle\boldsymbol{\beta}_j^{(1)}\|_1}{\lambda M^2 d\sqrt{d}} \\
&\le \|\triangle\boldsymbol{\beta}_j^{(1)}\|_1 (\frac{1}{\lambda M\sqrt{d/2}} + \frac{D}{\lambda M^2 d\sqrt{d}}),
\end{aligned}
$$

where $D = \max\{|\hat{\beta}_{mj}| : |\hat{\beta}_{mj}| \neq 0\}$. Therefore, there exists a small enough δ', if $\|\triangle\boldsymbol{\beta}_j^{(1)}\|_1 < \delta'$, we have $\|\hat{\boldsymbol{h}}' - \hat{\boldsymbol{h}}\|_1 + \|\hat{\boldsymbol{\xi}}' - \hat{\boldsymbol{\xi}}\|_1 < \delta$, then, due to the local maximality,

$$Q_3(\lambda, \hat{\boldsymbol{\beta}} + \triangle\boldsymbol{\beta}^{(1)}) = Q_2(\lambda, \hat{\boldsymbol{h}}', \hat{\boldsymbol{\xi}}') \le Q_2(\lambda, \hat{\boldsymbol{h}}, \hat{\boldsymbol{\xi}}) = Q_3(\lambda, \hat{\boldsymbol{\beta}}).$$

Next we show $Q_3(\lambda, \hat{\boldsymbol{\beta}} + \triangle\boldsymbol{\beta}^{(1)} + \triangle\boldsymbol{\beta}^{(2)}) \le Q_3(\lambda, \hat{\boldsymbol{\beta}} + \triangle\boldsymbol{\beta}^{(1)})$. By the mean value theorem,

we have

$$Q_3(\lambda, \hat{\boldsymbol{\beta}} + \triangle\boldsymbol{\beta}^{(1)} + \triangle\boldsymbol{\beta}^{(2)}) - Q_3(\lambda, \hat{\boldsymbol{\beta}} + \triangle\boldsymbol{\beta}^{(1)}) = (\triangle\boldsymbol{\beta}^{(2)})^T \nabla \ell(\check{\boldsymbol{\beta}}) - \lambda \sum_{j=1}^{p} \|\triangle\boldsymbol{\beta}_j^{(2)}\|_{\frac{1}{2}}^{\frac{1}{2}}, \tag{23}$$

where $\check{\boldsymbol{\beta}}$ is a vector between $\hat{\boldsymbol{\beta}} + \triangle\boldsymbol{\beta}^{(1)} + \triangle\boldsymbol{\beta}^{(2)}$ and $\hat{\boldsymbol{\beta}} + \triangle\boldsymbol{\beta}^{(1)}$. Since $\|\triangle\boldsymbol{\beta}^{(2)}\|_1 < \delta'$ is small enough, the second term plays a major role in (23) and is larger than the first term, hence we have

$$Q_3(\lambda, \hat{\boldsymbol{\beta}} + \triangle\boldsymbol{\beta}^{(1)} + \triangle\boldsymbol{\beta}^{(2)}) \le Q_3(\lambda, \hat{\boldsymbol{\beta}} + \triangle\boldsymbol{\beta}^{(1)}).$$

Overall, we can conclude that there exists a small enough $\delta' > 0$, if $\|\triangle\boldsymbol{\beta}\|_1 < \delta'$, then

$$Q_3(\lambda, \hat{\boldsymbol{\beta}} + \triangle\boldsymbol{\beta}) \le Q_3(\lambda, \hat{\boldsymbol{\beta}}),$$

which means that $\hat{\boldsymbol{\beta}}$ is a local maximizer of $Q_3(\lambda, \boldsymbol{\beta})$. Similarly, we can show that if $\hat{\boldsymbol{\beta}}$ is a local maximizer of $Q_3(\lambda, \boldsymbol{\beta})$, and if we let

$$(\hat{\boldsymbol{h}}, \hat{\boldsymbol{\xi}}) = \begin{cases} \hat{h}_j = 0, \hat{\boldsymbol{\xi}}_j = \mathbf{0}, & if\ \hat{\boldsymbol{\beta}}_j = \mathbf{0}, \\ \hat{h}_j = \lambda \|\hat{\boldsymbol{\beta}}_j\|_{1/2}^{1/2}, \hat{\boldsymbol{\xi}}_j = \frac{\hat{\boldsymbol{\beta}}_j}{\lambda \|\hat{\boldsymbol{\beta}}_j\|_{1/2}^{1/2}}, & if\ \hat{\boldsymbol{\beta}}_j \neq \mathbf{0}, \end{cases}$$

then $(\hat{\boldsymbol{h}}, \hat{\boldsymbol{\xi}})$ is a local maximizer of $Q_2(\lambda, \boldsymbol{h}, \boldsymbol{\xi})$. We omit the detailed proof process here.

Proof of Theorem 2

By the Theorem 1, we can see that under certain conditions, the meta-Half method and the $L_{1/2}$ regularization method are equivalent, so we only need to study the theoretical properties of the $L_{1/2}$ regularization method. According to the Theorem 1 in Xu et al.[1], Fan [2] has shown the sparsity and unbiasedness of the $L_{1/2}$ regularizer. Knight [3] studied the asymptotic normal property of the L_1 type regularizers, in essence, he has proved that the $L_{1/2}$ regularizer has the Oracle property. Therefore, the $L_{1/2}$ regularizer possesses sparsity, unbiasedness and Oracle properties. In conclusion, theorem 2 is proved.

Bibliography

[1] Zong Ben Xu, Hai Zhang, Yao Wang, Xiang Yu Chang, and Yong Liang. $l_{1/2}$ regularization. *Science China Information Sciences*, 53(6):1159–1169, 2010.

[2] Jianqing Fan, Heng Peng, et al. Nonconcave penalized likelihood with a diverging number of parameters. *The Annals of Statistics*, 32(3):928–961, 2004.

[3] Keith Knight, Wenjiang Fu, et al. Asymptotics for lasso-type estimators. *The Annals of statistics*, 28(5):1356–1378, 2000.
